# Heterogeneous brain dynamic functional connectivity patterns in first‐episode drug‐naive patients with major depressive disorder

**DOI:** 10.1002/hbm.26266

**Published:** 2023-03-15

**Authors:** Rixing Jing, Xiao Lin, Zengbo Ding, Suhua Chang, Le Shi, Lin Liu, Qiandong Wang, Juanning Si, Mingxin Yu, Chuanjun Zhuo, Jie Shi, Peng Li, Yong Fan, Lin Lu

**Affiliations:** ^1^ School of Instrument Science and Opto‐Electronics Engineering Beijing Information Science and Technology University Beijing China; ^2^ Peking University Sixth Hospital, Peking University Institute of Mental Health, NHC Key Laboratory of Mental Health (Peking University), National Clinical Research Center for Mental Disorders (Peking University Sixth Hospital), Chinese Academy of Medical Sciences Research Unit Peking University Beijing China; ^3^ National Institute on Drug Dependence and Beijing Key Laboratory on Drug Dependence Research Peking University Beijing China; ^4^ Beijing Key Laboratory of Applied Experimental Psychology, National Demonstration Center for Experimental Psychology Education (Beijing Normal University), Faculty of Psychology Beijing Normal University Beijing China; ^5^ Key Laboratory of Real‐Time Tracing of Brain Circuits of Neurology and Psychiatry (RTBNB_Lab), Tianjin Fourth Centre Hospital, Tianjin Medical University Affiliated Tianjin Fourth Centre Hospital Nankai University Affiliated Fourth Hospital Tianjin China; ^6^ Department of Radiology, Perelman School of Medicine University of Pennsylvania Philadelphia Pennsylvania USA; ^7^ Peking‐Tsinghua Center for Life Sciences and PKU‐IDG/McGovern Institute for Brain Research Peking University Beijing China

**Keywords:** dynamic functional connectivity pattern, first‐episode drug‐naive with major depressive disorder, heterogeneity, machine learning, normative model

## Abstract

It remains challenging to identify depression accurately due to its biological heterogeneity. As people suffering from depression are associated with functional brain network alterations, we investigated subtypes of patients with first‐episode drug‐naive (FEDN) depression based on brain network characteristics. This study included data from 91 FEDN patients and 91 matched healthy individuals obtained from the International Big‐Data Center for Depression Research. Twenty large‐scale functional connectivity networks were computed using group information guided independent component analysis. A multivariate unsupervised normative modeling method was used to identify subtypes of FEDN and their associated networks, focusing on individual‐level variability among the patients for quantifying deviations of their brain networks from the normative range. Two patient subtypes were identified with distinctive abnormal functional network patterns, consisting of 10 informative connectivity networks, including the default mode network and frontoparietal network. 16% of patients belonged to subtype I with larger extreme deviations from the normal range and shorter illness duration, while 84% belonged to subtype II with weaker extreme deviations and longer illness duration. Moreover, the structural changes in subtype II patients were more complex than the subtype I patients. Compared with healthy controls, both increased and decreased gray matter (GM) abnormalities were identified in widely distributed brain regions in subtype II patients. In contrast, most abnormalities were decreased GM in subtype I. The informative functional network connectivity patterns gleaned from the imaging data can facilitate the accurate identification of FEDN‐MDD subtypes and their associated neurobiological heterogeneity.

## Graphical abstract

Dynamic connectivity patterns were adopted to characterize the brain networks. A multivariate unsupervised normative modeling method was used to identify informative brain network patterns and subtypes of FEND patients. The subtypes showed significant differences in both functional connectivity and brain structural anatomy.

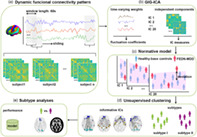

## 1 INTRODUCTION

According to the *Diagnostic and Statistical Manual of Mental Disorders, 5th Edition* (DSM‐5), the diagnosis of major depressive disorder (MDD) is made when a patient has any 5 out of 9 symptoms, resulting in dozens of symptom combinations (Goldberg, 2011). MDD is a heterogeneous syndrome varying significantly in illness progression, treatment response, genetics, and neurobiology. Genetic studies have highlighted MDD heterogeneity in differential polygenic risk profiles (Jermy, Glanville, Coleman, Lewis, & Vassos, 2021). The heterogeneity of MDD patients makes it difficult to develop personalized treatments and identify diagnostic biomarkers. To disentangle the heterogeneity in MDD, various attempts have been made to define subtypes based on clinical characteristics (Dunlop & Mayberg, 2017; Huibers et al., 2015). However, clinical features‐based classification is not robust because of the need for a solid theoretical background and neurobiological underpinnings (Drysdale et al., 2017). And also, the clinical symptoms change over time (Schaakxs et al., 2018). The present study aimed to identify subtypes of MDD based on neuroimaging data using machine learning methods.

Recent studies have identified disruptions in functional connectivity (FC) in specific brain networks in MDD (Brakowski et al., 2017; Rolls et al., 2018). Several brain networks were revealed to mediate depressive symptoms, including the default mode network (DMN) and salience brain network (Guo et al., 2014; Manoliu et al., 2013). However, the existing studies yielded inconsistent results in MDD (Runia et al., 2022). For instance, while an earlier study found that MDD was related to increased FC within the DMN (Qin et al., 2015), opposite results were reported by another study (C. Yan et al., 2019). Studies also suggested that the FC was influenced by the treatment. A longitude study found that abnormal FC for MDD patients disappeared after treatment in the posterior DMN, but persisted in the anterior DMN (Li et al., 2013). A similar study reported that 30% of the discriminative FCs were changed towards normal after antidepressant treatment (Qin et al., 2015). This inconsistency may result from the heterogeneity in the patients included in different studies, such as clinical symptoms, illness duration, and medical conditions. Specific patterns of functional brain networks might characterize distinct groups within MDD. Data‐driven approaches to explore the MDD subtypes based on neurobiological traits are promising (Loo, Jonge, Romeijn, Kessler, & Schoevers, 2012). Notably, a data‐driven clustering method has shown promising performance in grouping chronic, medicated MDD patients into four neurophysiological subtypes based on functional network measures (Drysdale et al., 2017). Two types of medication‐naive MDD significantly differed in symptom profiles and brain functional patterns were also identified in a previous study (Wang et al., 2021). A previous study revealed that abnormalities in brain networks in MDD patients could be blurred or hidden by the heterogeneity of the MDD clinical subgroups and brain plasticity may introduce a recovery effect to the abnormal network patterns seen in patients with a relatively short term of illness but unobservable in patients with long term of the illness (Xu et al., 2021). Moreover, a recent study revealed that no significant differences were observed between the chronic, medicated MDD patients and the first‐episode drug‐naive MDD patients (FEDN‐MDD) though both groups were significantly different from healthy controls in their dynamic functional connectivity patterns (Long et al., 2020).

Though many MDD studies assumed that the brain FC remains stationary during the scanning period, brain FC fluctuates over time could provide potentially new information on the brain function (Demirtaş et al., 2016; Hutchison et al., 2013; Mokhtari, Akhlaghi, Simpson, Wu, & Laurienti, 2019; Tu et al., 2019) and recent studies used sliding time window method found that temporal variability and characteristic temporal path length were significantly correlated with depression severity in MDD patients (Kaiser et al., 2016). Several studies have also found increased temporal fluctuations in the DMN (Demirtaş et al., 2016; Kaiser et al., 2016; Long et al., 2020), and one study reported the opposite result (Zhu et al., 2020). Most of these studies selected regions of interest based on previous studies (Cullen et al., 2009; Lui et al., 2011); in contrast, a whole brain analysis would be more objective and least biased to experience.

On the other hand, it has been shown that conventional group difference methods are not equipped to effectively characterize individual differences within groups (Marquand et al., 2019). Normative modeling is an alternative yet powerful means for characterizing normal variation of healthy controls and elucidating individual patients’ deviation from the controls (Bethlehem et al., 2020; Lv et al., 2020; Marquand, Rezek, Buitelaar, & Beckmann, 2016; Shan et al., 2022; Zabihi et al., 2019). Using normative modeling, researchers found that autism patients displayed highly individualized deviations in their cortical thickness, and the deviations were correlated with the severity of repetitive behaviors (Zabihi et al., 2019). Another brain structure study also revealed high heterogeneity in patients with schizophrenia (Lv et al., 2020).

**FIGURE 1:**
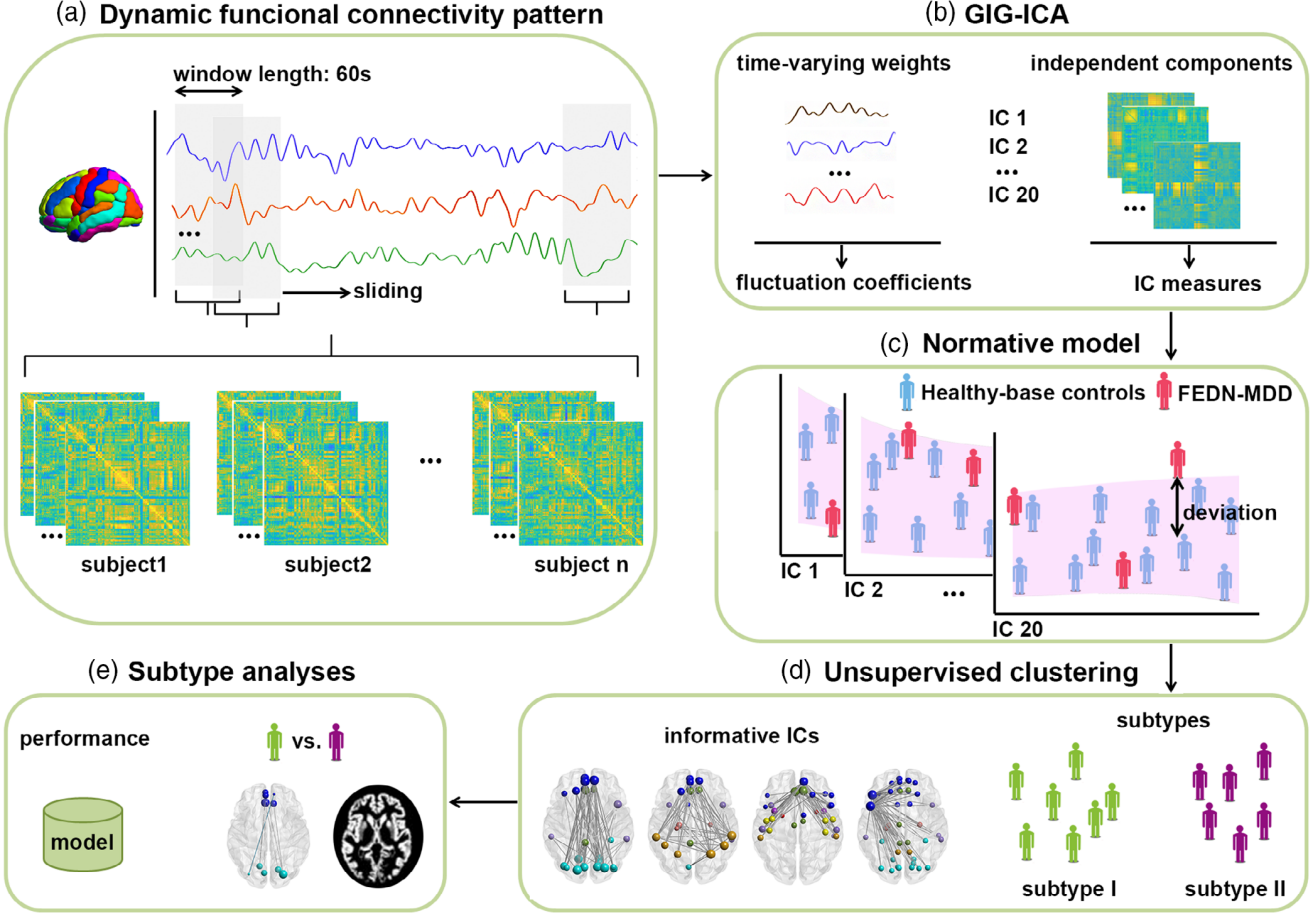
Methodological overview. (A) Resting‐state fMRI data were collected to create dynamic functional connectivity patterns based on the AAL atlas using a sliding time window method. (B) Healthy individuals from an independent dataset were used to compute the subject‐specific networks at the group level, and the back reconstruction algorithm of GIG‐ICA was used to compute inherent independent components (ICs) with the time‐varying weights of the remaining participants. (C) For the characteristics of healthy‐base controls (blue dots), the normative models were estimated between any characteristics and age to identify the normative distribution. Then, the deviations of each FEDN‐MDD individual (red dots) were quantified based on these normative models. (D) The deviations of each FEDN‐MDD individual were used as features to implement clustering analysis and identify the informative ICs. (E) Comparisons were performed on the clinical, functional connectivity and gray matters across the groups.

To elucidate MDD heterogeneity and identify robust MDD subtypes based on their functional magnetic resonance imaging (fMRI) data, we adopted the normative modeling method in conjunction with a data‐driven unsupervised method to characterize personalized functional connectivity patterns of FEDN‐MDD patients and identify FEDN‐MDD subtypes, as illustrated in Figure 1. Specifically, we used a data‐driven approach, group information‐guided independent component analysis (GIG‐ICA) (Du et al., 2017), to compute dynamic functional connectivity measures of individual subjects, including 91 FEDN‐MDD patients and 91 matched healthy controls. Then, to identify dynamic functional connectivity patterns for characterizing individual FEDN‐MDD, the personalized dynamic functional connectivity measures were analyzed using a normative modeling method. Finally, a clustering method was adopted to define subtypes based on dynamic functional connectivity patterns of the FEDN‐MDD. The differences between FEDN‐MDD subtypes were analyzed on both brain morphological and clinical measures, demonstrating their biological and clinical validity.

## 2 MATERIALS AND METHODS

### 2.1 Standard protocol approvals, registrations, and patient consent

In this study, we employed data from International Big‐Data Center for Depression Research (IBCDR), which was collected in China (The data is available by request from any qualified investigator. Website: http://yanlab.psych.ac.cn/IBCDR) (Deng et al., 2022; Ding et al., 2022). The data use agreement was attached as supplementary material. Deidentified and anonymized data were contributed from studies approved by local Institutional Review Boards. All study participants provided written informed consent at their local institution. Full details on the datasets and preprocessing pipeline have been described previously (C. Yan et al., 2019). Resting‐state fMRI was acquired at each site (TR = 2000 ms, TE = 30 ms, Flip Angle = 90°, number of volumes ≥210, opened eye) and was preprocessed using the DPARSF software with a standardized protocol (http://www.rfmri.org/DPARSF). According to the information provided by IBCDR, we included subjects for statistical analyses through the following criteria: (1) patients with first‐episode drug‐naive (FEDN) depression disorders; (2) subjects with age in the range of 18 to 50; (3) subjects with less head motion (mean FD < = 0.5 mm or max FD < = 2 mm). To avoid the center effect, 164 healthy individuals and 91 FEDN‐MDD patients (60 females) from the same center were included.

To prevent bias from these unmatched data, we used the matchIt function in R (https://www.rdocumentation.org/packages/MatchIt/versions/4.4.0/topics/matchit) to select the comparable group from the healthy controls as the healthy‐base group for the patients on the following characteristic: sex, age, and educational levels. Another independent sample (125 control individuals, 71 females) from the other two centers with similar data acquisition was a healthy‐ICA group to create the group‐level components as guidance information in ICA. Acquisition parameters of the dataset were shown in the supplementary materials (Table S1).

### 2.2 Dynamic functional connectivity pattern and feature extraction

For each subject, we computed whole‐brain dynamic functional connectivity matrices based on *m* (m = 90) ROIs from the automated anatomical labeling (AAL) template using a sliding time window method (window length = 60s, step size = 1) (Allen et al., 2014).

GIG‐ICA was adopted to the dynamic functional connectivity matrices to extract the subject‐specific independent components (ICs) and the corresponding time‐varying weights reflecting the variability of the ICs (Du et al., 2017). First, we chose the healthy‐ICA group to compute the ICs at the group level. Then these group‐level ICs were used as guidance information to calculate subject‐specific ICs of the FEDN‐MDD and healthy‐base controls. The number of IC was empirically determined to be 20. Finally, the dynamic functional connectivity pattern of each subject was represented by 20 IC measures (global efficiency coefficient of each IC) and 20 fluctuation coefficients (average change of the frame‐wise variation of each IC's time‐varying weights) (Figure 1B). The details of the approach are shown in the supplemental material.

### 2.3 Normative modeling and estimation of the feature deviations

After obtaining the 20 IC measures and 20 fluctuation coefficients of each healthy‐base control, we built the second‐order polynomial regression normative model (see supplementary materials) on the relationship between age and these 40 features, respectively. We estimated the 50^th^ (median) percentile with bootstrapping confidence intervals to quantify the extent of deviations for any FEDN‐MDD patient from the normative model. To avoid bias in the model, we used the range between the 5^th^ and 95^th^ percentiles for a given age as a scaling coefficient to normalize the z‐scores of the feature deviations. Thus, each FEDN‐MDD patient of the same age was positioned and a z‐score for quantifying each feature was created from the normative range as follows:

$$z_{\mathrm{jf}}^{i}=\frac{\left|{C.\text{real}}_{\mathrm{jf}}^{i}-{C.50^{\mathrm{th}}}_{jf}^{i}\right|}{\left|{C.95^{\mathrm{th}}}_{\mathrm{jf}}^{i}-{C.5^{\mathrm{th}}}_{\mathrm{jf}}^{i}\right|}$$

$$j=1,2,\ldots ,20,f=\left\{\text{ICmeasures},\text{fluctuation coefficients}\right\}$$

For individuals with index *i*, $z_{\mathrm{jf}}^{i}$ was the normalized deviation value for feature *f* of IC_*j*_. C.real was the real value of the feature, and $C.5^{\mathrm{th}}$, $C.50^{\mathrm{th}}$ and $C.95^{\mathrm{th}}$ were the predictions in 5^th^, 50^th^ and 95^th^ percentiles for the same age respectively.

To assess generalization, we applied 10‐fold cross‐validation to build an aggregation normative model for each feature (details in supplementary materials). The average value of the 10 individual z scores yielded from the normative aggregation model was the final feature deviation for each individual. Thus, each patient was characterized by a 40‐dimension feature vector concatenating 20 *IC measures’ deviations* and 20 *fluctuation coefficients’ deviations*.

### 2.4 A multivariate unsupervised method to identify the informative features

To investigate the importance of the features and remove the redundancy, a forward selection technique based on the k‐means algorithm was used to select features. The selected features were informative in exploring the homogeneous subgroups in the FEDN‐MDD cohort.

Specifically, the forward feature selection procedure was carried out 100 runs repeatedly to avoid any bias in the clustering. Since different features might be selected in different runs due to the random selection of initial centers in the k‐means algorithm, the selected informative features of FEDN‐MDD were identified as those with higher frequency. The details of the approach, including the optimal number of clusters were shown in the supplemental material. The number of clusters in k‐means was eventually identified as k = 2, considering the performance and the limitation of the sample size.

Similarly, a 100‐repeated clustering procedure based on the selected informative features was applied to cluster the FEDN‐MDD patients, and the overall performance was quantified based on these 100 runs. Finally, each FEDN‐MDD patient was given 100 individual clustering labels that yielded 100 repeated runs. A certainty measure to evaluate the clustering reliability for each subject based on 100 repeated runs. Notably, the clustering certainty was measured by certainty value ($\text{certainty}=\max_{i=1,\ldots ,k}\left(\frac{n_{i}}{n}\right)$, where $n_{i}$ is the number of labels *i* and *n* is the total number of repeated runs). A higher value indicated higher clustering reliability and vice versa. We excluded 2 subjects with lower clustering certainty (<0.6) in post hoc analyses.

The FEDN‐MDD patients’ labels were obtained by $i=\arg\max_{i=1,\ldots ,k}\left(\frac{n_{i}}{n}\right)$ as a robust measure to characterize the heterogeneity of FEDN‐MDD, and two subtypes of FEDN‐MDD were identified.

Pseudo two sample t‐tests were conducted to explore the functional connectivity differences of the ICs between healthy‐based controls and subtypes of FEDN‐MDD using statistical nonparametric mapping software (SnPM13, http://warwick.ac.uk/snpm; permutation tests n = 5000, age, sex and education as covariates).

### 2.5 Voxel‐based morphometry comparisons

To explore if functional changes were associated with structural alterations, voxel‐based morphometry (VBM) analysis was carried out to compare gray matter (GM) maps of FEDN patients and healthy subjects. Pseudo two sample t‐tests were adopted to compare GM maps between healthy‐base controls and subtypes of FEDN‐MDD using permutation tests (n = 10000, age, sex, education and total incranial volume (TIV) as covariates).

## 3 RESULTS

### 3.1 Demographic characteristic

Sample characteristics are provided in Table 1. We used the matching method to match the healthy controls from the patients on the following characteristic: sex, age, and educational levels. The included healthy‐based group did not differ in sex, age, and educational levels from the FEDN‐MDD.

**TABLE 1:** Demographic data

	FEDN‐MDD^a^	Controls		p‐values
		Healthy‐ICA group	Healthy‐base Group^a^	
*N*	91	125	91	
Age (years)	34.80 ± 9.49	31.11 ± 8.50	32.49 ± 11.79	.15
Gender (male)	60	71	65	.52
Illness duration (months)	29.97 ± 44.01	—	—	—
Education (years)	11.60 ± 3.33	14.31 ± 2.59	12.43 ± 3.55	.11
HAMD	22.03 ± 4.06	—	—	—
HAMA	23.92 ± 7.79	—	—	—
FD				
Mean	0.07 ± 0.04	0.06 ± 0.04	0.07 ± 0.03	.45
Max	0.40 ± 0.37	0.37 ± 0.37	0.44 ± 0.38	.17

Abbreviations: FD, frame‐wise displacement; FEDN‐MDD, first‐episode drug‐naive major depression disorders; HAMD, Hamilton Depression Scale; HAMA, Hamilton Anxiety Scale.

*Note*: The index of head movement, calculated from the derivatives of the six rigid‐body realignment parameters estimated during standard volume realignment. Scores: Mean ± SD is shown.

^a^ The comparison was conducted on these two groups.

### 3.2 Informative features for FEDN‐MDD

**FIGURE 2:**
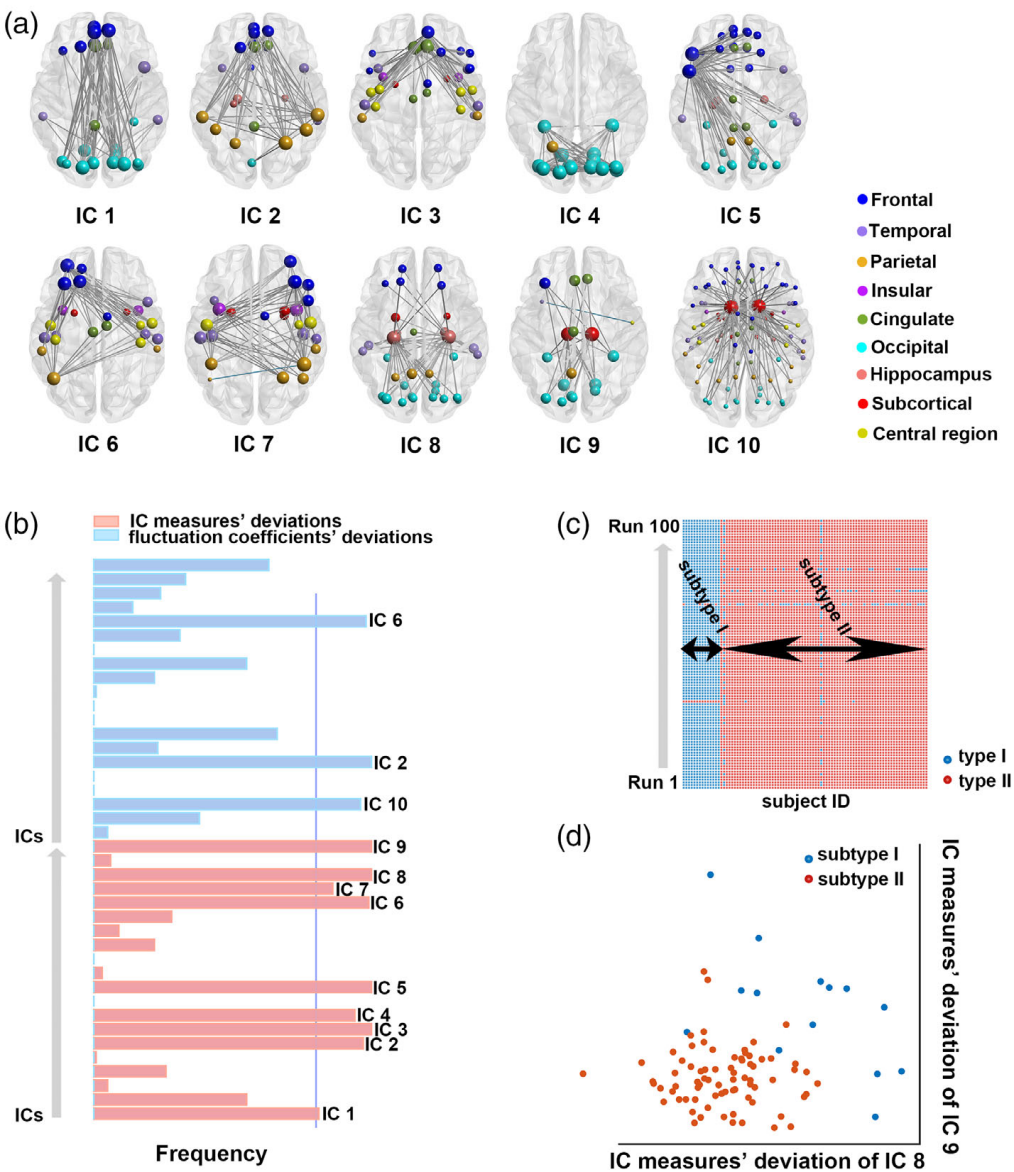
The results of the clustering approach. (a) The informative ICs identified by the wrapper unsupervised feature selection method. (b) The frequency of the ICs that were selected in the repeated cross‐validation experiments. ICs selected with the highest frequencies (>0.8) were deemed informative ICs. (c) The type labels for each patient across 100 clustering runs. (d) A schematic demonstration of the clustering results based on two‐dimensional features.

Twelve features from 10 ICs were selected as informative features to cluster the FEDN‐MDD. These selected features primarily consisted of IC measures of IC 1 to IC 9, and fluctuation coefficients of IC 2, IC 6, and IC 10 (see Figure 2B). These informative ICs involved the brain network in the default mode network (DMN) (IC 1), the frontoparietal networks (IC 2), the ACC‐hub network (IC 3), the visual network (IC 4), the language‐related network (IC 5), the bilateral executive control network (IC 6 and IC 7), the hippocampus‐hub network (IC 8), the thalamus‐hub network (IC 9), and the caudate‐hub network (IC 10, for details, see Figure 2A). As shown in Figure 2C, across the 100 runs, the labels of each patient were consistent, with only two patients with lower label consistency excluded (clustering certainty <0.8). The high consistency reflected the clustering reliability for each subject. As shown in the two‐dimensional plot in Figure 2D, the plot contained 2 clusters arranged in the first two dimensions.

### 3.3 Clustering analysis and clinical symptom comparisons in two subtypes of FEDN‐MDD

**FIGURE 3:**
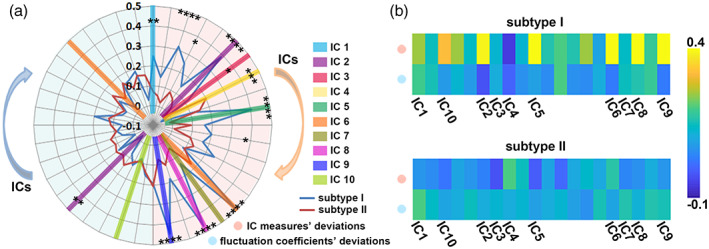
Clustering centers across two subtypes. (A) A radar plot represented the center of the features’ deviations of each subtype, and the significant differences were computed in each feature (*p < .05, **p < .01, ***p < .001, ****p < .0001). (B) The center of the features deviations of each subtype was represented by a heatmap. The top row of the pattern delineated 20 connectivity network measures’ deviations, with the bottom row delineating another 20 fluctuation coefficients’ deviations.

**FIGURE 4:**
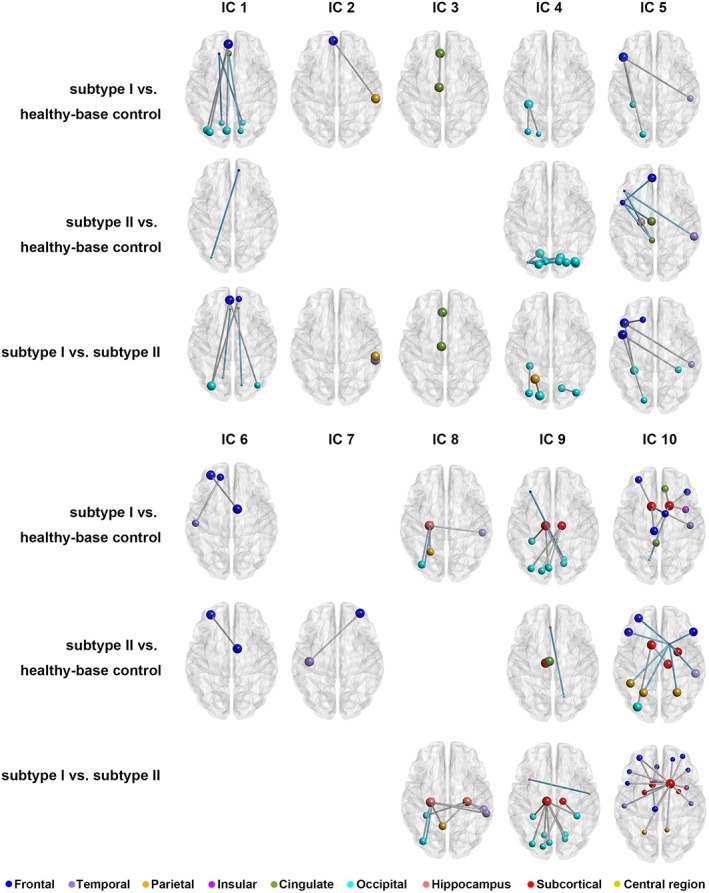
The group differences in functional connectivity in the informative ICs across three groups (*p* < .01). For a comparison between X versus Y, silver color: X > Y, cyan color: X < Y.

The proposed multivariate unsupervised approach identified two subtypes of the FEDN‐MDD, with 14 patients as subtype I and 75 patients as subtype II. The mean silhouette index (Rousseeuw, 1987) of the clustering was 0.461, and the instability of the cluster centers (De Mulder, 2014) was 4.08. The mean stability of the clustering was 0.97. There was a clear distinction between the identified two subtypes on the 12 informative features (see Figure 3A). The average deviations of each feature for each subtype were shown in Figure 3B, and two subtypes showed significant differences in deviation characteristics, especially the 20 IC measures’ deviations. To some extent, the deviations pattern of subtype I was more significant than subtype II, and this pattern was limited to the informative IC measures’ deviations. A comparison of intra‐IC functional connectivity of 10 informative ICs showed a significant difference in each brain network among the three groups (5000 permutation tests, *p* < .01). The differences are shown in Figure 4. There were more abnormalities across all informative networks in the subtype I patients than in the subtype II patients. Compared with the healthy controls, the subtype I patients showed increased and decreased functional connectivities between the frontal and occipital cortex in the DMN (IC 1). And the subtype II patients mostly showed decreased functional connectivity in this particular brain network.

The severity of depression and anxiety did not differ between subtypes. However, the illness duration in subtype II (22.1 ± 23.92) patients was longer than in subtype I (11.75 ± 12.09) (*p* = .03, Table 2). In addition, the subtype II patients had fewer extreme deviations in the IC measures and fluctuation coefficients from the normal range.

**TABLE 2:** Participant demographics of two FEDN‐MDD subtypes

	Subtype I	Subtype II	p‐values
*N*	14	75	
Age (years)	35.36 ± 8.12	34.73 ± 9.77	.82
Gender (female)	10	49	.66
Illness duration (months)	11.75 ± 12.09	22.123 ± 23.92	**.03**
Education (years)	12.14 ± 2.54	11.51 ± 3.46	.51
HAMD	22.14 ± 5.39	22.07 ± 3.85	.67
HAMA	22.71 ± 7.19	23.09 ± 7.88	.67
FD			
Mean	0.08 ± 0.03	0.06 ± 0.03	.93
Max	0.39 ± 0.26	0.40 ± 0.39	.15

### 3.4 Voxel‐based morphometry comparisons

**FIGURE 5:**
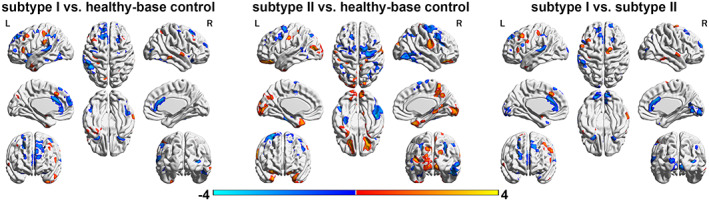
The group differences in gray matter in the informative ICs across three groups (*p* < .05, cluster size >200). For a comparison between X versus Y, warm color: X > Y, cold color: X < Y.

Considering the well‐documented relationship between the illness duration and the brain structural changes (Kanaan et al., 2009), we also analyzed the differences in whole‐brain GM between the two subtypes. Compared to healthy controls, the subtype II patients showed increased GM (mainly in the middle frontal regions and several posterior brain regions) and decreased GM (mainly in temporal and superior frontal regions) distributed widely in the brain. In contrast, subtype I patients, mainly showed decreased GM in the temporal cortex and anterior cingulate gyrus (see Figure 5).

## 4 DISCUSSION

The present study suggested that FEDN‐MDD patients might be a heterogeneous group, mainly classified into two subtypes by a multivariate unsupervised method based on normative models. Two subtypes of patients showed different illness durations, GM abnormalities, and functional connectivity alterations in several informative connectivity networks. The subtype I patients were those with more extreme deviations in the functional characteristics of brain networks, and also with shorter illness durations. Subtype II patients had fewer extreme functional deviations with longer illness durations. As for functional connectivity, the subtype I patients had more abnormalities than healthy controls, and the subtype II patients showed fewer differences compared to healthy controls. Intriguingly, compared with healthy controls, the subtype II patients showed increased and decreased GM. In contrast, the subtype I showed mostly decreased GM. These results herein indicated that the altered brain function of patients became more convergent as the illness duration increased. However, the range of the illness duration is relatively limited, since the illness of duration of subtype II patients was around 22 months and the duration of subtype I patients was 11 months.

Instead of the traditional case–control studies that tried to localize the differences between two groups, the normative model provides estimated individual deviations from the normal range. Mainly, quantile regression aims to seek the median or any other percentiles and enables statistical analysis to describe the full range of normal variation compared to conventional regression seeking the mean of the variable to be predicted. In the present study, we constructed a second‐order normative model on the features of the brain networks established through GIG‐ICA. The results revealed that most participants fit the normative model well, suggesting that most were in the normal range of variation. This result was consistent with previous studies conducted on patients with schizophrenia (Alexander‐Bloch et al., 2014) and autism disorders (Bethlehem et al., 2020). The more patients with extreme deviations from the normative range in FEDN patients suggested that patients with MDD might be more heterogeneous. Some of the largest neuroimaging meta‐analyses have revealed a neuropathological characteristic of profound functional brain changes, significantly affecting all cortical regions (Gray, Müller, Eickhoff, & Fox, 2020; Hamilton et al., 2012). While this provided an accurate group‐level characterization, our findings suggested that such profound and widespread abnormalities did not represent all the individuals with FEDN‐MDD. Pooling individuals from different subtypes and testing for case–control differences in the pooled cohort is likely to yield a group‐average combination of the distinct changes associated with each subtype. Clustering patients into subtypes helped parse the heterogeneity of FEDN and cut across conventional diagnostic boundaries. Combining the normative model with a data‐driven clustering approach illustrated the occurrence of separable subgroups in FEDN, with distinct brain characteristics.

Ten of the whole brain functional networks are informative at clustering the subtypes of FEDN‐MDD. The DMN and frontoparietal brain networks are the most frequently reported abnormal brain network in MDD. A previous study found two neural subtypes of MDD associated with different dysfunctional DMN connectivity patterns (Liang et al., 2020). Another study that used the machine learning approach found that the most discriminative features are distributed in the visual network, somatomotor network, attention network, limbic network, frontoparietal network, and DMN (B. Yan et al., 2020). Of the two subtypes we identified, when compared to healthy controls, the subtype II patients showed significantly decreased connectivity in DMN. In general, the subtype I patients showed more increased connectivities, compared to healthy controls, especially in the DMN and the limbic systems (the hippocampus‐hub, the caudate‐hub and the thalamus‐hub networks, for details, see Figure 4). The increased and decreased functional connectivity in subtype I and subtype II patient could explain the inconsistent results in previous studies. The limbic system comprises key emotional areas that have been reported to be increased‐ (Jiao et al., 2020), decreased‐(Goya‐Maldonado et al., 2016), or normally(Dai, Zhou, Xu, & Zuo, 2019) connected in depression. And similar inconsistency was also reported in the within‐network FC of the executive control network (ECN), as excessive (Yao et al., 2019) or deficient (Stange et al., 2017) FC have been reported in depression. In a prior study, MDD patients were divided into two biotypes according to the functional connectivity changes in the DMN, one patient subgroup was characterized by hyperconnectivity within the DMN, and one subgroup had decreased connectivity (Liang et al., 2020). In the present study, the pattern was similar but not limited to the DMN, for most brain networks, with one group showing hyper‐connectivity while the other showed hypo‐connectivity compared to healthy controls. In addition to distinguishing subtype features, some abnormal network patterns (e.g., the decreased functional connectivity between the dorsal and lateral frontal cortex in IC 5) were shared across the two patient subgroups. A previous study compared the functional connectivity of the thalamus in 31 first‐episode MDD and 28 healthy controls and found that the patients showed significant abnormal thalamus functional connectivity. This abnormality is correlated with the severity of symptoms (Kang et al., 2018). In the present study, abnormal functional connectivities are mainly found in subtype I patients. In contrast, subtype II patients had a distribution that was less deviated from healthy controls and had relatively longer illness durations. Importantly, the severity of depression and anxiety did not differ between subtypes, indicating that our subtype delineation did not merely separate mild and more severely ill patients. In a recent study, reduced GM volumes were consistently found in two cohorts of clinically diagnosed MDD participants (Hellewell et al., 2019). The structural changes in subtype II patients were more complex than the subtype I patients, since there are both increased and decreased GM abnormalities distributed widely in brain regions in subtype II patients, and most of the abnormalities in subtype I were decreased GM. Several previous studies indicated that the volumes or the FA were reversely correlated with the illness duration (Serra‐Blasco et al., 2013; Zhao et al., 2021). In the present study, the subtype II patients have relatively longer illness duration, and more complex brain structural abnormalities. A recently published study divided MDD patients into two groups based on the illness duration, and they found that the network difference in MDD from HCs was confined to those patients with shorter illness durations (Xu et al., 2021). In this study, the difference in illness duration between the two subtypes is not significant enough to get a positive conclusion on the relationship between illness duration and structural/functional brain changes. However, the present results posed an essential opportunity for future studies to investigate structural and functional correlations with illness duration.

Several limitations of this study should be considered. First, our study included treatment‐naive patients. The medication's effects on functional connectivity and symptoms did not confound the data analysis. However, it is unclear whether our findings apply to MDD groups with multiple prior episodes and active antidepressant therapies. Second, in certain respects of the method, the ideal choice for the clustering algorithm would be a powerful clustering method capable of detecting clusters of very different types. However, k‐means was used in this study, which seeks compact clusters whose time complexity is only linear in the number of data items. Furthermore, the empirical number of ICs should be estimated automatically. Third, this data was from a cross‐section study, which lacks treatment information; the value of this clustering on treatment prediction remains to be known. Forth, the generalizability of the current finding is limited since there are only 14 patients as subtype I in a specific FEDN cohort.

In the present study, we applied the normative model to connectivity network measures and fluctuation coefficients of the brain inherent networks. Combined with the data‐driven multivariate clustering method, we recognized the patient cohort into two subtypes. Two subtypes of patients showed different illness durations and significantly different functional connectivities with GM alterations in several essential brain networks. Our findings provided insight into FEDN depression heterogeneity in biological terms, and represented a promising step toward categorical subtype recognition and personalized treatments.

## AUTHOR CONTRIBUTIONS

Rixing Jing: Major role in the acquisition of data; Analysis, or interpretation of data; Xiao Lin: Drafting/revision of the manuscript for content; Study concept or design; Zengbo Ding: Drafting/revision of the manuscript, including writing for content; Suhua Chang: Drafting/revision of the manuscript; Le Shi: Drafting/revision of the manuscript for content; Lin Liu: Drafting/revision of the manuscript; Qiandong Wang: Analysis or interpretation of data; Drafting/revision of the manuscript; Juanning Si: Drafting/revision of the manuscript; Mingxin Yu: Drafting/revision of the manuscript; Chuanjun Zhuo: Drafting/revision of the manuscript; Jie Shi: Drafting/revision of the manuscript; Interpretation of data; Peng Li: Major role in the acquisition of data; Interpretation of data; Yong Fan: Drafting/revision of the manuscript; Analysis or interpretation of data; Lin Lu: Drafting/revision of the manuscript, Study concept or design.

## CONFLICT OF INTEREST

The authors report no disclosures relevant to the manuscript.

## FUNDING STATEMENT

This work was supported in part by Beijing Natural Science Foundation (No.7214299, 7212141), the National High Technology Research and Development Program of China (No. 2019YFA0706201), the R&D Program of Beijing Municipal Education Commission (No. KM202011232007, No. KM202011232008), the National Natural Science Foundation of China (No. 82101566), and Beijing Natural Science Foundation (No. 4214080).

## Supporting information


**Data S1:** Supporting Information

## Data Availability

The imaging and clinical data used in the present study were obtained from the International Big‐Data Center for Depression Research (for details, http://yanlab.psych.ac.cn/IBCDR). The data and code are available upon request from any qualified investigator.

## References

[hbm26266-bib-0001] Alexander‐Bloch, A. F. , Reiss, P. T. , Rapoport, J. , McAdams, H. , Giedd, J. N. , Bullmore, E. T. , & Gogtay, N. (2014). Abnormal cortical growth in schizophrenia targets normative modules of synchronized development. Biological Psychiatry, 76(6), 438–446. 24690112 10.1016/j.biopsych.2014.02.010 PMC4395469

[hbm26266-bib-0002] Allen, E. A. , Damaraju, E. , Plis, S. M. , Erhardt, E. B. , Eichele, T. , & Calhoun, V. D. (2014). Tracking whole‐brain connectivity dynamics in the resting state. Cerebral Cortex, 3, 663–676. 10.1093/cercor/bhs352 PMC3920766 23146964

[hbm26266-bib-0003] Bethlehem, R. A. I. , Seidlitz, J. , Romero‐Garcia, R. , Trakoshis, S. , Dumas, G. , & Lombardo, M. V. (2020). A normative modelling approach reveals age‐atypical cortical thickness in a subgroup of males with autism spectrum disorder. Communications Biology, 3(1), 486. 32887930 10.1038/s42003-020-01212-9 PMC7474067

[hbm26266-bib-0004] Brakowski, J. , Spinelli, S. , Dörig, N. , Bosch, O. G. , Manoliu, A. , Holtforth, M. G. , & Seifritz, E. (2017). Resting state brain network function in major depression ‐ depression symptomatology, antidepressant treatment effects, future research. Journal of Psychiatric Research, 92, 147–159. 28458140 10.1016/j.jpsychires.2017.04.007

[hbm26266-bib-0005] Cullen, K. R. , Gee, D. G. , Klimes‐Dougan, B. , Gabbay, V. , Hulvershorn, L. , Mueller, B. A. , Camchong, J. , Bell, C. J. , Houri, A. , Kumra, S. , Lim, K. O. , Castellanos, F. X. , & Milham, M. P. (2009). A preliminary study of functional connectivity in comorbid adolescent depression. Neuroscience Letters, 460(3), 227–231. 19446602 10.1016/j.neulet.2009.05.022 PMC2713606

[hbm26266-bib-0006] Dai, L. , Zhou, H. , Xu, X. , & Zuo, Z. (2019). Brain structural and functional changes in patients with major depressive disorder: A literature review. PeerJ, 7, e8170. 31803543 10.7717/peerj.8170 PMC6886485

[hbm26266-bib-0007] De Mulder, W. (2014). Instability and cluster stability variance for real clusterings. Information Sciences, 260, 51–63.

[hbm26266-bib-0008] Demirtaş, M. , Tornador, C. , Falcón, C. , López‐Solà, M. , Hernández‐Ribas, R. , Pujol, J. , MenchóŁn, J. M. , Ritter, P. , Cardoner, N. , Soriano‐Mas, C. , & Deco, G. (2016). Dynamic functional connectivity reveals altered variability in functional connectivity among patients with major depressive disorder. Human Brain Mapping, 37(8), 2918–2930. 27120982 10.1002/hbm.23215 PMC5074271

[hbm26266-bib-0009] Deng, K. , Yue, J.‐H. , Xu, J. , Ma, P.‐P. , Chen, X. , Li, L. , Bai, T. J. , Bo, Q. J. , Cao, J. , Chen, G. M. , Chen, N. X. , Chen, W. , Cheng, C. , Cui, X. L. , Duan, J. , Fang, Y. R. , Gong, Q. Y. , Guo, W. B. , Hou, Z. H. , … Cheng, Y. Q. (2022). Impaired robust interhemispheric function integration of depressive brain from REST‐meta‐MDD database in China. Bipolar Disorders, 24(4), 400–411. 34606159 10.1111/bdi.13139

[hbm26266-bib-0010] Ding, Y. D. , Chen, X. , Chen, Z. B. , Li, L. , Li, X. Y. , Castellanos, F. X. , Bai, T. J. , Bo, Q. J. , Cao, J. , Chang, Z. K. , Chen, G. M. , Chen, N. X. , Chen, W. , Cheng, C. , Cheng, Y. Q. , Cui, X. L. , Duan, J. , Fang, Y. R. , Gong, Q. Y. , … Guo, W. B. (2022). Reduced nucleus accumbens functional connectivity in reward network and default mode network in patients with recurrent major depressive disorder. Translational Psychiatry, 12(1), 236. 35668086 10.1038/s41398-022-01995-x PMC9170720

[hbm26266-bib-0011] Drysdale, A. T. , Grosenick, L. , Downar, J. , Dunlop, K. , Mansouri, F. , Meng, Y. , Fetcho, R. N. , Zebley, B. , Oathes, D. J. , Etkin, A. , Schatzberg, A. F. , Sudheimer, K. , Keller, J. , Mayberg, H. S. , Gunning, F. M. , Alexopoulos, G. S. , Fox, M. D. , Pascual‐Leone, A. , Voss, H. U. , … Liston, C. (2017). Resting‐state connectivity biomarkers define neurophysiological subtypes of depression. Nature Medicine, 23(1), 28–38. 10.1038/nm.4246 PMC5624035 27918562

[hbm26266-bib-0012] Du, Y. , Pearlson, G. D. , Lin, D. , Sui, J. , Chen, J. , Salman, M. , Tamminga, C. A. , Ivleva, E. I. , Sweeney, J. A. , Keshavan, M. S. , Clementz, B. A. , Bustillo, J. , & Calhoun, V. D. (2017). Identifying dynamic functional connectivity biomarkers using GIG‐ICA: Application to schizophrenia, schizoaffective disorder, and psychotic bipolar disorder: Identify dynamic connectivity states via GIG‐ICA. Human Brain Mapping, 38(5), 2683–2708. 28294459 10.1002/hbm.23553 PMC5399898

[hbm26266-bib-0013] Dunlop, B. W. , & Mayberg, H. S. (2017). Neuroimaging advances for depression. Cerebrum, 2017, cer‐16–17. PMC6132047 30210664

[hbm26266-bib-0014] Goldberg, D. (2011). The heterogeneity of “major depression”. World Psychiatry, 10(3), 226–228. 21991283 10.1002/j.2051-5545.2011.tb00061.x PMC3188778

[hbm26266-bib-0015] Goya‐Maldonado, R. , Brodmann, K. , Keil, M. , Trost, S. , Dechent, P. , & Gruber, O. (2016). Differentiating unipolar and bipolar depression by alterations in large‐scale brain networks. Human Brain Mapping, 37(2), 808–818. 26611711 10.1002/hbm.23070 PMC6867444

[hbm26266-bib-0016] Gray, J. P. , Müller, V. I. , Eickhoff, S. B. , & Fox, P. T. (2020). Multimodal abnormalities of brain structure and function in major depressive disorder: A meta‐analysis of neuroimaging studies. The American Journal of Psychiatry, 177(5), 422–434. 32098488 10.1176/appi.ajp.2019.19050560 PMC7294300

[hbm26266-bib-0017] Guo, W. , Liu, F. , Zhang, J. , Zhang, Z. , Yu, L. , Liu, J. , Chen, H. , & Xiao, C. (2014). Abnormal default‐mode network homogeneity in first‐episode, drug‐naive major depressive disorder. PloS One, 9(3), e91102. 24609111 10.1371/journal.pone.0091102 PMC3946684

[hbm26266-bib-0018] Hamilton, J. P. , Etkin, A. , Furman, D. J. , Lemus, M. G. , Johnson, R. F. , & Gotlib, I. H. (2012). Functional neuroimaging of major depressive disorder: A meta‐analysis and new integration of base line activation and neural response data. The American Journal of Psychiatry, 169(7), 693–703. 22535198 10.1176/appi.ajp.2012.11071105 PMC11889638

[hbm26266-bib-0019] Hellewell, S. C. , Welton, T. , Maller, J. J. , Lyon, M. , Korgaonkar, M. S. , Koslow, S. H. , Williams, L. M. , Rush, A. J. , Gordon, E. , & Grieve, S. M. (2019). Profound and reproducible patterns of reduced regional gray matter characterize major depressive disorder. Translational Psychiatry, 9(1), 176. 31341158 10.1038/s41398-019-0512-8 PMC6656728

[hbm26266-bib-0021] Huibers, M. J. H. , Cohen, Z. D. , Lemmens, L. H. J. M. , Arntz, A. , Peeters, F. P. M. L. , Cuijpers, P. , & DeRubeis, R. J. (2015). Predicting optimal outcomes in cognitive therapy or interpersonal psychotherapy for depressed individuals using the personalized advantage index approach. PloS One, 10(11), e0140771. 26554707 10.1371/journal.pone.0140771 PMC4640504

[hbm26266-bib-0022] Hutchison, R. M. , Womelsdorf, T. , Allen, E. A. , Bandettini, P. A. , Calhoun, V. D. , Corbetta, M. , Della Penna, S. , Duyn, J. H. , Glover, G. H. , Gonzalez‐Castillo, J. , Handwerker, D. A. , Keilholz, S. , Kiviniemi, V. , Leopold, D. A. , de Pasquale, F. , Sporns, O. , Walter, M. , & Chang, C. (2013). Dynamic functional connectivity: Promise, issues, and interpretations. NeuroImage, 80, 360–378. 23707587 10.1016/j.neuroimage.2013.05.079 PMC3807588

[hbm26266-bib-0023] Jermy, B. S. , Glanville, K. P. , Coleman, J. , Lewis, C. M. , & Vassos, E. (2021). Exploring the genetic heterogeneity in major depression across diagnostic criteria. Molecular Psychiatry, 12(26), 7337–7345. 10.1038/s41380-021-01231-w PMC8872976 34290369

[hbm26266-bib-0024] Jiao, K. , Xu, H. , Teng, C. , Song, X. , Xiao, C. , Fox, P. T. , Zhang, N. , Wang, C. , & Zhong, Y. (2020). Connectivity patterns of cognitive control network in first episode medication‐naive depression and remitted depression. Behavioural Brain Research, 379, 112381. 31770543 10.1016/j.bbr.2019.112381

[hbm26266-bib-0025] Kaiser, R. H. , Whitfield‐Gabrieli, S. , Dillon, D. G. , Goer, F. , Beltzer, M. , Minkel, J. , Smoski, M. , Dichter, G. , & Pizzagalli, D. A. (2016). Dynamic resting‐state functional connectivity in major depression. Neuropsychopharmacology, 41(7), 1822–1830. 26632990 10.1038/npp.2015.352 PMC4869051

[hbm26266-bib-0026] Kanaan, R. , Barker, G. , Brammer, M. , Giampietro, V. , Shergill, S. , Woolley, J. , Picchioni, M. , Toulopoulou, T. , & McGuire, P. (2009). White matter microstructure in schizophrenia: Effects of disorder, duration and medication. British Journal of Psychiatry, 194(3), 236–242. 10.1192/bjp.bp.108.054320 PMC2802507 19252154

[hbm26266-bib-0027] Kang, L. , Zhang, A. , Sun, N. , Liu, P. , Yang, C. , Li, G. , Liu, Z. , Wang, Y. , & Zhang, K. (2018). Functional connectivity between the thalamus and the primary somatosensory cortex in major depressive disorder: A resting‐state fMRI study. BMC Psychiatry, 18(1), 339. 30340472 10.1186/s12888-018-1913-6 PMC6194586

[hbm26266-bib-0028] Li, B. , Liu, L. , Friston, K. J. , Shen, H. , Wang, L. , Zeng, L.‐L. , & Hu, D. (2013). A treatment‐resistant default mode subnetwork in major depression. Biological Psychiatry, 74(1), 48–54. 23273724 10.1016/j.biopsych.2012.11.007

[hbm26266-bib-0029] Liang, S. , Deng, W. , Li, X. , Greenshaw, A. J. , Wang, Q. , Li, M. , Ma, X. , Bai, T. J. , Bo, Q. J. , Cao, J. , Chen, G. M. , Chen, W. , Cheng, C. , Cheng, Y. Q. , Cui, X. L. , Duan, J. , Fang, Y. R. , Gong, Q. Y. , Guo, W. B. , … Li, T. (2020). Biotypes of major depressive disorder: Neuroimaging evidence from resting‐state default mode network patterns. NeuroImage. Clinical, 28, 102514. 33396001 10.1016/j.nicl.2020.102514 PMC7724374

[hbm26266-bib-0030] Long, Y. , Cao, H. , Yan, C. , Chen, X. , Li, L. , Castellanos, F. X. , Bai, T. , Bo, Q. , Chen, G. , Chen, N. , Chen, W. , Cheng, C. , Cheng, Y. , Cui, X. , Duan, J. , Fang, Y. , Gong, Q. , Guo, W. , Hou, Z. , … Liu, Z. (2020). Altered resting‐state dynamic functional brain networks in major depressive disorder: Findings from the REST‐meta‐MDD consortium. NeuroImage: Clinical, 26, 102163. 31953148 10.1016/j.nicl.2020.102163 PMC7229351

[hbm26266-bib-0031] Lui, S. , Wu, Q. , Qiu, L. , Yang, X. , Kuang, W. , Chan, R. C. , Huang, X. , Kemp, G. J. , Mechelli, A. , & Gong, Q. (2011). Resting‐state functional connectivity in treatment‐resistant depression. The American Journal of Psychiatry, 168(6), 642–648. 21362744 10.1176/appi.ajp.2010.10101419

[hbm26266-bib-0032] Lv, J. , Di Biase, M. , Cash, R. F. H. , Cocchi, L. , Cropley, V. L. , Klauser, P. , Tian, Y. , Bayer, J. , Schmaal, L. , Cetin‐Karayumak, S. , Rathi, Y. , Pasternak, O. , Bousman, C. , Pantelis, C. , Calamante, F. , & Zalesky, A. (2020). Individual deviations from normative models of brain structure in a large cross‐sectional schizophrenia cohort. Molecular Psychiatry, 26(7), 3512–3523. 32963336 10.1038/s41380-020-00882-5 PMC8329928

[hbm26266-bib-0033] Manoliu, A. , Meng, C. , Brandl, F. , Doll, A. , Tahmasian, M. , Scherr, M. , Schwerthöffer, D. , Zimmer, C. , Förstl, H. , Bäuml, J. , Riedl, V. , Wohlschläger, A. M. , & Sorg, C. (2013). Insular dysfunction within the salience network is associated with severity of symptoms and aberrant inter‐network connectivity in major depressive disorder. Frontiers in Human Neuroscience, 7, 930. 24478665 10.3389/fnhum.2013.00930 PMC3896989

[hbm26266-bib-0034] Marquand, A. F. , Kia, S. M. , Zabihi, M. , Wolfers, T. , Buitelaar, J. K. , & Beckmann, C. F. (2019). Conceptualizing mental disorders as deviations from normative functioning. Molecular Psychiatry, 24(10), 1415–1424. 31201374 10.1038/s41380-019-0441-1 PMC6756106

[hbm26266-bib-0035] Marquand, A. F. , Rezek, I. , Buitelaar, J. , & Beckmann, C. F. (2016). Understanding heterogeneity in clinical cohorts using normative models: Beyond case‐control studies. Biological Psychiatry, 80(7), 552–561. 26927419 10.1016/j.biopsych.2015.12.023 PMC5023321

[hbm26266-bib-0036] Mokhtari, F. , Akhlaghi, M. I. , Simpson, S. L. , Wu, G. , & Laurienti, P. J. (2019). Sliding window correlation analysis: Modulating window shape for dynamic brain connectivity in resting state. NeuroImage, 189, 655–666. 30721750 10.1016/j.neuroimage.2019.02.001 PMC6513676

[hbm26266-bib-0037] Qin, J. , Shen, H. , Zeng, L.‐L. , Jiang, W. , Liu, L. , & Hu, D. (2015). Predicting clinical responses in major depression using intrinsic functional connectivity. NeuroReport, 26(12), 675–680. 26164454 10.1097/WNR.0000000000000407

[hbm26266-bib-0038] Rolls, E. T. , Cheng, W. , Gong, W. , Qiu, J. , Zhou, C. , Zhang, J. , Lv, W. , Ruan, H. , Wei, D. , Cheng, K. , Meng, J. , Xie, P. , & Feng, J. (2018). Functional connectivity of the anterior cingulate cortex in depression and in health. Cerebral Cortex, 29(8), 3717–3630. 10.1093/cercor/bhy236 30418547

[hbm26266-bib-0039] Rousseeuw, P. J. (1987). Silhouettes: A graphical aid to the interpretation and validation of cluster analysis. Journal of Computational and Applied Mathematics, 20, 53–65.

[hbm26266-bib-0040] Runia, N. , Yücel, D. E. , Lok, A. , de Jong, K. , Denys, D. A. J. P. , van Wingen, G. A. , & Bergfeld, I. O. (2022). The neurobiology of treatment‐resistant depression: A systematic review of neuroimaging studies. Neuroscience & Biobehavioral Reviews, 132, 433–448. 34890601 10.1016/j.neubiorev.2021.12.008

[hbm26266-bib-0041] Schaakxs, R. , Comijs, H. C. , Lamers, F. , Kok, R. M. , Beekman, A. T. F. , & Penninx, B. W. J. H. (2018). Associations between age and the course of major depressive disorder: A 2‐year longitudinal cohort study. The Lancet. Psychiatry, 5(7), 581–590. 29887519 10.1016/S2215-0366(18)30166-4

[hbm26266-bib-0042] Serra‐Blasco, M. , Portella, M. J. , Gómez‐Ansón, B. , de Diego‐Adelio, J. , Vives‐Gilabert, Y. , Puigdemont, D. , Granell, E. , Santos, A. , Alvarez, E. , & Pérez, V. (2013). Effects of illness duration and treatment resistance on grey matter abnormalities in major depression. The British Journal of Psychiatry: The Journal of Mental Science, 202, 434–440. 23620451 10.1192/bjp.bp.112.116228

[hbm26266-bib-0043] Shan, X. , Uddin, L. Q. , Xiao, J. , He, C. , Ling, Z. , Li, L. , Huang, X. , Chen, H. , & Duan, X. (2022). Mapping the heterogeneous brain structural phenotype of autism Spectrum disorder using the normative model. Biological Psychiatry, 91(11), 967–976. 35367047 10.1016/j.biopsych.2022.01.011

[hbm26266-bib-0044] Stange, J. P. , Bessette, K. L. , Jenkins, L. M. , Peters, A. T. , Feldhaus, C. , Crane, N. A. , Ajilore, O. , Jacobs, R. H. , Watkins, E. R. , & Langenecker, S. A. (2017). Attenuated intrinsic connectivity within cognitive control network among individuals with remitted depression: Temporal stability and association with negative cognitive styles. Human Brain Mapping, 38(6), 2939–2954. 28345197 10.1002/hbm.23564 PMC5426983

[hbm26266-bib-0045] Tu, Y. , Fu, Z. , Zeng, F. , Maleki, N. , Lan, L. , Li, Z. , Park, J. , Wilson, G. , Gao, Y. , Liu, M. , Calhoun, V. , Liang, F. , & Kong, J. (2019). Abnormal thalamocortical network dynamics in migraine. Neurology, 92(23), 2706–2716. 10.1212/WNL.0000000000007607 PMC6556096 31076535

[hbm26266-bib-0020] van Loo, H. M. , de Jonge, P. , Romeijn, J. W. , Kessler, R. C. , & Schoevers, R. A. (2012). Data‐driven subtypes of major depressive disorder: A systematic review. BMC Medicine, 10, 156. 23210727 10.1186/1741-7015-10-156 PMC3566979

[hbm26266-bib-0046] Wang, Y. , Tang, S. , Zhang, L. , Bu, X. , Lu, L. , Li, H. , Gao, Y. , Hu, X. , Kuang, W. , Jia, Z. , Sweeney, J. A. , Gong, Q. , & Huang, X. (2021). Data‐driven clustering differentiates subtypes of major depressive disorder with distinct brain connectivity and symptom features. The British Journal of Psychiatry, 219(5), 606–613. 35048829 10.1192/bjp.2021.103

[hbm26266-bib-0047] Xu, D. , Xu, G. , Zhao, Z. , Sublette, M. E. , Miller, J. M. , & Mann, J. J. (2021). Diffusion tensor imaging brain structural clustering patterns in major depressive disorder. Human Brain Mapping, 42(15), 5023–5036. 34312935 10.1002/hbm.25597 PMC8449115

[hbm26266-bib-0048] Yan, B. , Xu, X. , Liu, M. , Zheng, K. , Liu, J. , Li, J. , Wei, L. , Zhang, B. , Lu, H. , & Li, B. (2020). Quantitative identification of major depression based on resting‐state dynamic functional connectivity: A machine learning approach. Frontiers in Neuroscience, 14, 191. 32292322 10.3389/fnins.2020.00191 PMC7118554

[hbm26266-bib-0049] Yan, C. , Chen, X. , Li, L. , Castellanos, F. X. , Bai, T. , Bo, Q. , Cao, J. , Chen, G. M. , Chen, N. X. , Chen, W. , Cheng, C. , Cheng, Y. Q. , Cui, X. L. , Duan, J. , Fang, Y. R. , Gong, Q. Y. , Guo, W. B. , Hou, Z. H. , Hu, L. , … Zang, Y. F. (2019). Reduced default mode network functional connectivity in patients with recurrent major depressive disorder. Proceedings of the National Academy of Sciences of the United States of America, 116(18), 9078–9083. 30979801 10.1073/pnas.1900390116 PMC6500168

[hbm26266-bib-0050] Yao, Z. , Shi, J. , Zhang, Z. , Zheng, W. , Hu, T. , Li, Y. , Yu, Y. , Zhang, Z. , Fu, Y. , Zou, Y. , Zhang, W. , Wu, X. , & Hu, B. (2019). Altered dynamic functional connectivity in weakly‐connected state in major depressive disorder. Clinical Neurophysiology, 130(11), 2096–2104. 31541987 10.1016/j.clinph.2019.08.009

[hbm26266-bib-0051] Zabihi, M. , Oldehinkel, M. , Wolfers, T. , Frouin, V. , Goyard, D. , Loth, E. , Charman, T. , Tillmann, J. , Banaschewski, T. , Dumas, G. , Holt, R. , Baron‐Cohen, S. , Durston, S. , Bölte, S. , Murphy, D. , Ecker, C. , Buitelaar, J. K. , Beckmann, C. F. , & Marquand, A. F. (2019). Dissecting the heterogeneous cortical anatomy of autism Spectrum disorder using normative models. Biological Psychiatry. Cognitive Neuroscience and Neuroimaging, 4(6), 567–578. 30799285 10.1016/j.bpsc.2018.11.013 PMC6551348

[hbm26266-bib-0052] Zhao, W. , Zhu, D. , Zhang, Y. , Zhang, C. , Zhang, B. , Yang, Y. , Zhu, J. , & Yu, Y. (2021). Relationship between illness duration, corpus callosum changes, and sustained attention dysfunction in major depressive disorder. Quantitative Imaging in Medicine and Surgery, 11(7), 2980–2982. 34249628 10.21037/qims-20-970 PMC8250000

[hbm26266-bib-0053] Zhu, D. , Yang, Y. , Zhang, Y. , Wang, C. , Wang, Y. , Zhang, C. , Zhao, W. , & Zhu, J. (2020). Cerebellar‐cerebral dynamic functional connectivity alterations in major depressive disorder. Journal of Affective Disorders, 275, 319–328. 32734925 10.1016/j.jad.2020.06.062

