# Supplementary material for: Heterogeneous brain dynamic functional connectivity patterns in first‐episode drug‐naive patients with major depressive disorder
Source: Hum Brain Mapp. 2023 Mar 15;44(8):3112–22. doi: 10.1002/hbm.26266 (PMC10171501; doi:10.1002/hbm.26266)

February 12, 2023, a Revised Article for *Human Brain Mapping [No.* HBM-22-1047.R1*]*

**SUPPLEMENTARY MATERIAL**

**Heterogeneous brain dynamic functional connectivity patterns in first-episode drug-naive patients with major depressive disorder**

Rixing Jing^1, #^, Xiao Lin^2, #^ , Zengbo Ding^3^, Suhua Chang^2^, Le Shi^2^, Lin Liu^3^, Qiandong Wang^4^, Juanning Si^1^, Mingxin Yu^1^, Chuanjun Zhuo^5^, Jie Shi^3^, Peng Li^2,*^, Yong Fan^6,*^, Lin Lu^2, 3, 7,*^

^1^ School of Instrument Science and Opto-Electronics Engineering, Beijing Information Science and Technology University, Beijing 100192, China

^2^ Peking University Sixth Hospital, Peking University Institute of Mental Health, NHC Key Laboratory of Mental Health (Peking University), National Clinical Research Center for Mental Disorders (Peking University Sixth Hospital), Chinese Academy of Medical Sciences Research Unit (No.2018RU006), Peking University, Beijing 100191, China

^3^ National Institute on Drug Dependence and Beijing Key Laboratory on Drug Dependence Research, Peking University, Beijing 100191, China

^4^ Beijing Key Laboratory of Applied Experimental Psychology, National Demonstration Center for Experimental Psychology Education (Beijing Normal University), Faculty of Psychology, Beijing Normal University, Beijing 100875, China.

^5^ Key Laboratory of Real-Time Tracing of Brain Circuits of Neurology and Psychiatry (RTBNB_Lab), Tianjin Fourth Centre Hospital, Tianjin Medical University Affiliated Tianjin Fourth Centre Hospital, Nankai University Affiliated Fourth Hospital, Tianjin 300142, China

^6^ Department of Radiology, Perelman School of Medicine, University of Pennsylvania, Philadelphia, PA 19104, USA

^7^ Peking-Tsinghua Center for Life Sciences and PKU-IDG/McGovern Institute for Brain Research, Beijing 100191, China

**Running title:** Brain networks identified two FEDN MDD subtypes

# Equal Author Contribution:

Rixing Jing and Xiao Lin contributed equally as the first authors.

*Corresponding author:

Lin Lu, MD, Ph.D., Institute of Mental Health and Peking University Sixth Hospital, 51 Huayuanbei Road, Beijing, 100191, China.

Tel: +86-10-82805308; Fax: +86-10-62032624; E-mail: [linlu@bjmu.edu.cn](mailto:linlu@bjmu.edu.cn)

Yong Fan, Ph.D., Department of Radiology, Perelman School of Medicine, University of Pennsylvania, Philadelphia, PA 19104, USA.

Tel: +1-215-746-4065; Fax: +1-215-573-1811; Email: yong.fan@ieee.org

Peng Li, Ph.D., Institute of Mental Health and Peking University Sixth Hospital, 51 Huayuanbei Road, Beijing, 100191, China.

Tel: +86-10-82803704; Fax: +86-10-62032624; E-mail: [lipeng1986@bjmu.edu.cn](mailto:lipeng1986@bjmu.edu.cn)

**Manuscript information:** 5 Figures, 2 Tables, 1 Supplementary Material

**Methods**

**Dynamic functional connectivity pattern and feature extraction**

Group information-guided ICA (GIG-ICA) was applied to extract independent components (ICs) as connectivity networks from the dynamic functional patterns. The input of the GIG-ICA was whole-brain time-varying connectivity matrices computed between all pairs of regions (m=90) of the AAL template using a sliding time window method as the dynamic functional patterns. The outputs of the GIG-ICA were ICs and the corresponding time-varying weights reflecting the variability of ICs. The detailed calculation steps are as follows: 1. All connectivity matrices can be converted to a vector containing m×(m-1)/2=4005 elements, and then the time-varying connectivity patterns of each subject can be represented by a window-by-connectivity matrix (size: w×4005, w is the number of windows). 2. The window-direction concatenated dynamic connectivity patterns of all subjects can be represented by X (size: (w·n) ×4005, n is the number of subjects in the healthy-ICA group). 3. The Infomax algorithm was applied to the Fisher-transformed X to estimate the group-level components with a two-step principal component analysis for data reduction. 4. Based on these identified group-level components and the individual-level dynamic functional connectivity patterns from patients and healthy-base controls, GIG-ICA was used to estimate the corresponding subject-specific independent components with time-varying weights using a multiple-objective optimization function.

For each IC was a connectivity state/network, we computed the global efficiency coefficient based on IC, and the fluctuation coefficient was computed based on the IC’s time-varying weights.

**Table S1. Key acquisition parameters of the included data.**

| FEDN-MDD and Healthy-base Group | | | | | | | | | | |
| --- | --- | --- | --- | --- | --- | --- | --- | --- | --- | --- |
| Site number | No. of FEDN-MDD | No. of Healthy-base Group | Receive  (coil) | TR  (ms) | TE  (ms) | FA (degree) | Thickness/gap | Slice  number | Time  point | Voxel  size |
| 20 | 91 | 91 | 12 channel | 2000 | 30 | 90 | 3.0mm/1.0mm | 32 | 242 | 3.44*3.44*4.00 |
| Healthy-ICA Group | | | | | | | | | | |
| 1 | - | 67 | 32 channel | 2000 | 30 | 90 | 4.0 mm /0.8 mm | 30 | 210 | 3.28*3.28 *4.80 |
| 21 | - | 58 | 32 channel | 2000 | 30 | 90 | 3.5 mm /0.7 mm | 30 | 240 | 3.12*3.12 *4.20 |

**Normative modeling and estimation of the feature deviations**

*To identify the feature deviations, a quantile polynomial regression model was used to determine percentile curves as normative models Field (Lv et al., 2021).* Compared to conventional regression seeking the mean of the variable to be predicted, quantile regression aims to seek the median or any other percentiles. It enabled statistical analysis to describe the full range of normal variation. Polynomial regression, including the linear and quadratic models as two special cases, provides a methodological alternative to offer greater validity when evaluating the nature of informant-self discrepancies (Zhang, Zhang, & Wang, 2014).

To assess generalization, we applied 10-fold cross-validation to build an aggregation normative model. We repeatedly trained the polynomial regression model on 90% of the 91 healthy-base participants at different percentiles, withholding the remaining 10% for estimating generalization performance. This 10-fold cross-validation procedure yielded 10 normative models. Each of these models generated a normative distribution based on 3 ranges with 95% confidence intervals. Thus, each FEDN-MDD patient was given 10 individual z scores and the average value was the feature deviation. Finally, each patient was characterized by a 40-dimension feature vector concatenating *20 IC measures’ deviations* and *20 fluctuation coefficients’ deviations*.

**Unsupervised** **feature selection and clustering analysis**

A feature selection method that assesses feature utility with respect to an unsupervised clustering algorithm was used to identify informative features. In this study, the k-means algorithm was used to seek compact clusters. In certain respects, the ideal choice for the clustering algorithm would be a powerful clustering method capable of detecting clusters of very different types. In this study, the number of clusters was tuned (*k* = 2, 3, 4) to optimize the clustering performance firstly. The number of clusters in k-means was eventually identified as k = 2 considering the performance and the limitation of the sample. (Table S2).

**Table S2. The performance of the k-means algorithm**

| Number of clusters | 2 | 3 | 4 | 5 |
| --- | --- | --- | --- | --- |
| Silhouette Coefficient | 0.12 | 0.08 | 0.06 | 0.05 |
| Calinski-Harabaz Index | 5.71 | 4.66 | 4.00 | 3.62 |
| Instability of Cluster Centers | 15.24 | 10.36 | 7.98 | 7.01 |

We built clustering models on a subset of 40 features to explore the homogeneous subgroups in the FEDN-MDD cohort using the k-means algorithm. The k-means algorithm was based on similarity measures between subjects computed based on their features. A simplified forward selection technique was adopted to identify the subset of features with the best clustering performance using the following procedure. First, the forward selection algorithm built a k-means model upon each feature, the model’s performance was then estimated with 20-fold cross-validation so that each feature could be evaluated for its clustering performance. We chose the Silhouette index (SI) (Rousseeuw, 1987)and the instability of cluster centers (ICC) (De Mulder, 2014) as the measurements of clustering performance. Thus, the feature with the best SI and ICC was selected to be included in the clustering. Combining the first selected feature and one of the other features, a clustering model could be built upon two features. All models built on two features were then evaluated using the 20-fold cross-validation, and the features with the best performance were selected to be included in the clustering. This procedure was repeated to include more features in the clustering one by one until one single clustering model was built upon all available features. Finally, the combination of features with the overall best performance was chosen as the informative features.

**Results**

**Characterization of extreme deviations of IC measures and fluctuation coefficients**

Twenty reliable group-level IC were estimated from the fMRI data of healthy-ICA controls. As stated in the method section, for each group-level network, the corresponding subject-specific network was estimated for each subject using GIG-ICA. The *t*-value map was obtained by performing voxel-wise right-tailed one-sample *t*-tests on all subjects' networks in Supplementary Figure S1.

The validity of the normative models was established based on 10-fold cross-validation. We found that the ratio of healthy-base control with deviated global efficiency is about 3.85% (supra 1.81%, infra 2.03%). Meanwhile, 5.17% FEDN-MDD participants (supra 2.53%, infra 2.64%) showed extreme deviations of IC measures in at least one of the functional components. As for the fluctuation coefficients, the ratio of healthy controls with significant extreme deviations is 4.18% (supra 2.03%, infra 2.14%), and the ratio of patients is 5.35% (supra 2.30%, infra 3.05%). Then, we placed each participant in the typical distribution to identify alterations in individual cases with respect to the IC measures and fluctuation coefficients. In all of the participants, 91.2% of patients have higher certainty (certainty efficiency > 0.6, certainty efficiency was the maximum ratio of measures located in supra, infra or normal range in cross-validation procedure) in the normative modeling, with 8 FEDN-MDD subjects showing lower certainty in the modeling process. The deviant values were replaced with the average value of the whole participants for cleaning the dataset.

**Replication experiments**

We used the second-order polynomial regression model to capture a potential quadratic relationship, in addition to the linear one in our data. Compared with the simplest linear model, the second-order model introduced one more parameter. We also replaced the second-model with a linear model and re-analyzed the data. Two patients with lower label consistency were excluded in the regression analysis. Thus, the total number of subjects in the statistic is 89. The linear model-based results were similar to those derived from the second-order model. Specifically, similar subtypes were identified with 17 patients as subtype I and 72 patients as subtype II, as illustrated by the following confusion matrix, indicating that the second-order model obtained reasonably good results as the linear model.

**Table S3. The confusion matrix of the subtype label between polynomial regression and l**inear regression.

|  | | Polynomial Regression | | Sum |
| --- | --- | --- | --- | --- |
|  |  | Subtype I | Subtype II |  |
| Linear Regression | Subtype I | 13 | 4 | 17 |
|  | Subtype II | 1 | 71 | 72 |
| Sum | | 14 | 75 | 89 |

**References:**

De Mulder, W. (2014). Instability and cluster stability variance for real clusterings. *Information Sciences, 260*, 51-63.

Lv, J., Di Biase, M., Cash, R. F. H., Cocchi, L., Cropley, V. L., Klauser, P., . . . Zalesky, A. (2021). Individual deviations from normative models of brain structure in a large cross-sectional schizophrenia cohort. *Molecular Psychiatry, 26*(7), 3512-3523.

Rousseeuw, P. J. (1987). Silhouettes: A graphical aid to the interpretation and validation of cluster analysis. *Journal of Computational and Applied Mathematics, 20*, 53-65.

Tzourio-Mazoyer, N., Landeau, B., Papathanassiou, D., Crivello, F., Etard, O., Delcroix, N., . . . Joliot, M. (2002). Automated anatomical labeling of activations in SPM using a macroscopic anatomical parcellation of the MNI MRI single-subject brain. *NeuroImage, 15*(1), 273-289.

Zhang, T., Zhang, Q., & Wang, Q. (2014). Model detection for functional polynomial regression. *Computational Statistics & Data Analysis, 70*, 183-197.

**Figure S1.** All inherent independent components (IC) were yielded from GIG-ICA.


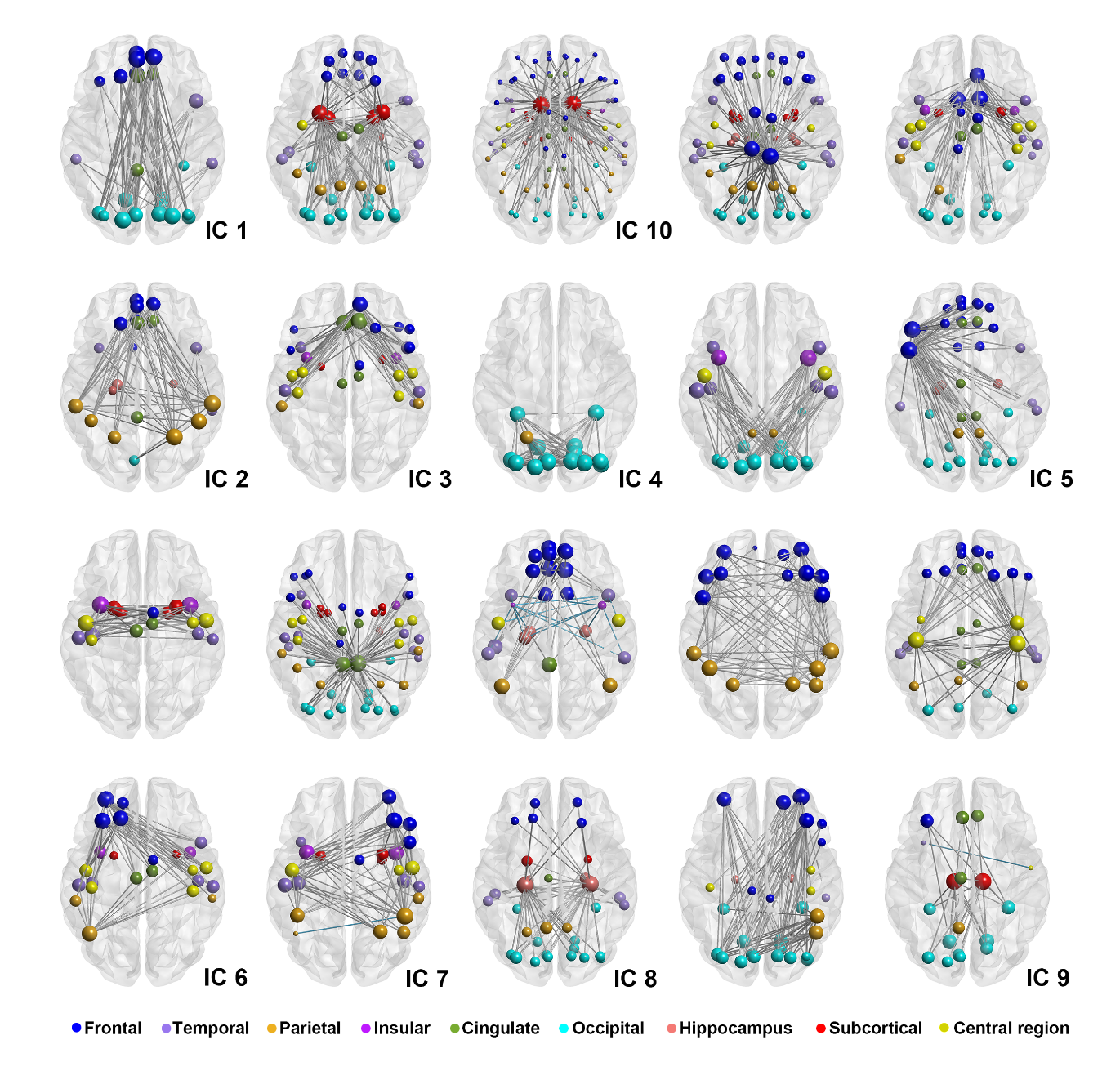

Supplement: Supplementary file 1 — Data S1: Supporting Information [file HBM-44-3112-s001.doc]
